# Fracability evaluation of the middle–upper Permian marine shale reservoir in well HD1, western Hubei area

**DOI:** 10.1038/s41598-023-40735-z

**Published:** 2023-08-31

**Authors:** Shengling Jiang, Qinghua Zhou, Yanju Li, Rili Yang

**Affiliations:** 1 School of New Energy, Longdong University, No. 45, Lanzhou Road, Xifeng District, Qingyang, 745000 Gansu China; 2 Oil and Gas Exploration and Production Branch, China HuaDian Green Energy, Co. Ltd., Beijing, 100160 China

**Keywords:** Solid Earth sciences, Energy science and technology, Engineering

## Abstract

Taking the HD1 well as the research target, through intensive core sampling, experimental test analysis, comprehensive logging interpretation and other methods, the fracability of shale reservoirs was discussed in this paper. The results show that the Gufeng–Dalong Formation in the HD1 well has a high organic carbon content, and the organic-rich shale developed in the lower part of the Gufeng and Dalong Formations with thicknesses of 30 m and 15 m, respectively. For the low-porosity and ultralow-permeability shale reservoir type, the natural fractures are undeveloped in the lower part of the Dalong Formation, with a lower linear density, while they are well developed in the Xiayao and Longtan Formations and the lower part of the Gufeng Formation, and the interlayer bedding fractures are relatively developed. The Gufeng–Dalong Formation shale also has a high mineral brittleness index (average of 44.5%), high static Young’s modulus (20–70 GPa), low static Poisson’s ratio (0.10–0.31), and high horizontal pressure difference coefficient of two phases (0.17–0.56). It is concluded that the shale reservoir is favorable for fracture development in the lower part of the Dalong Formation, with depths of 1249–1289.5 m, and the lower part of the Xiayao, Longtan and Gufeng Formations, with depths of 1300–1335.3 m. Overall, the fracable shale section with high brittleness and rock strength is beneficial to fracturing. However, the existence of a large number of shale bedding fractures increases the complexity of the fractures, and at the same time, it has a certain negative impact on fracture generation. Double wing fractures are easily formed because of the high two-phase horizontal pressure difference. Therefore, the leakage caused by shale bedding fractures and the influence on the fracture height and extension length should be considered comprehensively in fracturing design.

## Introduction

China has some of the most developed Permian strata in the world. The sedimentary types of Permian shales are diverse. Marine, marine–continental transitional and continental facies coexist^1^. The research on Permian shale gas mainly focuses on the Ordos Basin and its peripheral areas^2–5^, Qinshui Basin and its peripheral areas^6–8^, and Lower Yangtze platform area^9–12^, but different types of shale gas resources are mainly in marine–continental transitional facies. The middle–upper Permian shale in the Yangtze platform is widely developed, with thicknesses of 25–200 m, and the thickness center is in the Ziyang–Yibin–Luzhou area. The sedimentary facies are mainly open platform, tidal flat and lagoon facies, which are still classified as marine–continental transitional facies^13^. The middle–upper Permian marine shales are mainly distributed in the Sangzhi–Enshi–Hefeng–Jianshi area and Xiangfan–Jingzhou–Xianning area, belonging to the ancient Kaijiang–Liangping Basin, western Hubei Basin and northern basin^1,14,15^, with basin facies signaling a deep-water sedimentary environment. The middle–upper Permian marine shale is a set of high-quality source rocks that is rich in organic matter^16–18^.

The discovery of the Fuling shale gas field represented the first breakthrough in shale gas exploration of the Upper Ordovician Wufeng Formation–lower Silurian Longmaxi Formation in the Sichuan Basin^19–21^. The exploration of shale gas in the Western Hunan and Hubei area, Central Yangtze platform, has not yet made a breakthrough, but a large amount of research work has been carried out because of the continuous progress of exploration and the continuous accumulation of data. Exploration and research work mainly focuses on the lower Cambrian Niutitang Formation and Upper Ordovician Wufeng Formation–lower Silurian Longmaxi Formation shales^22–29^. There are relatively few studies on middle–upper Permian shale gas in the western Hunan and Hubei areas, and the exploration and research work mainly focuses on the geological conditions of shale development^30–32^, shale reservoir characteristics and preliminary evaluation of gas-bearing properties^33^. There are also relatively few studies on the fracability evaluation of shale reservoirs in western Hunan and Hubei based on literature investigations. Chen et al.^34^ discussed the fracability of black siliceous shale and carbonaceous shale in the middle and lower parts of the Niutitang Formation in northwestern Hunan based on the results of field outcrop tests by establishing a mathematical model for fracability evaluation. Wu et al.^35^ evaluated the effect of macro- and micro-parameters on the fracability of the lower Cambrian Niutitang shale of northwestern Hunan based on field investigations and sample analysis. Cui et al.^36^ quantitatively evaluated the shale reservoir fracability in the Baojing area, Hunan Province, based on the correlation analysis method and the analytic hierarchy process. Jiang et al.^37^ taking the HD1 well as the research target, evaluated the fracability of shale reservoirs in the lower Cambrian Niutitang Formation, northwestern Hunan. Li^38^ expounds the research progress on evaluation methods and factors influencing shale brittleness. Li et al.^39^ evaluated shale fracability based on whole-rock X-ray diffraction analysis, scanning electron microscopy, shale gas-bearing experiments, rock mechanical parameter tests and well logging and elemental logging data.

Accordingly, in this paper, we conduct a comprehensive study on the fracability evaluation of the fracturing properties of middle–upper Permian marine shale in the western Hunan and Hubei areas by core description and core sample test data combined with logging calculations, which are rarely used in research. Then, we select favorable fracturing intervals for Permian marine shale that is rich in organic matter and propose suggestions for later fracturing improvement.

## Geological setting

**Figure 1:**
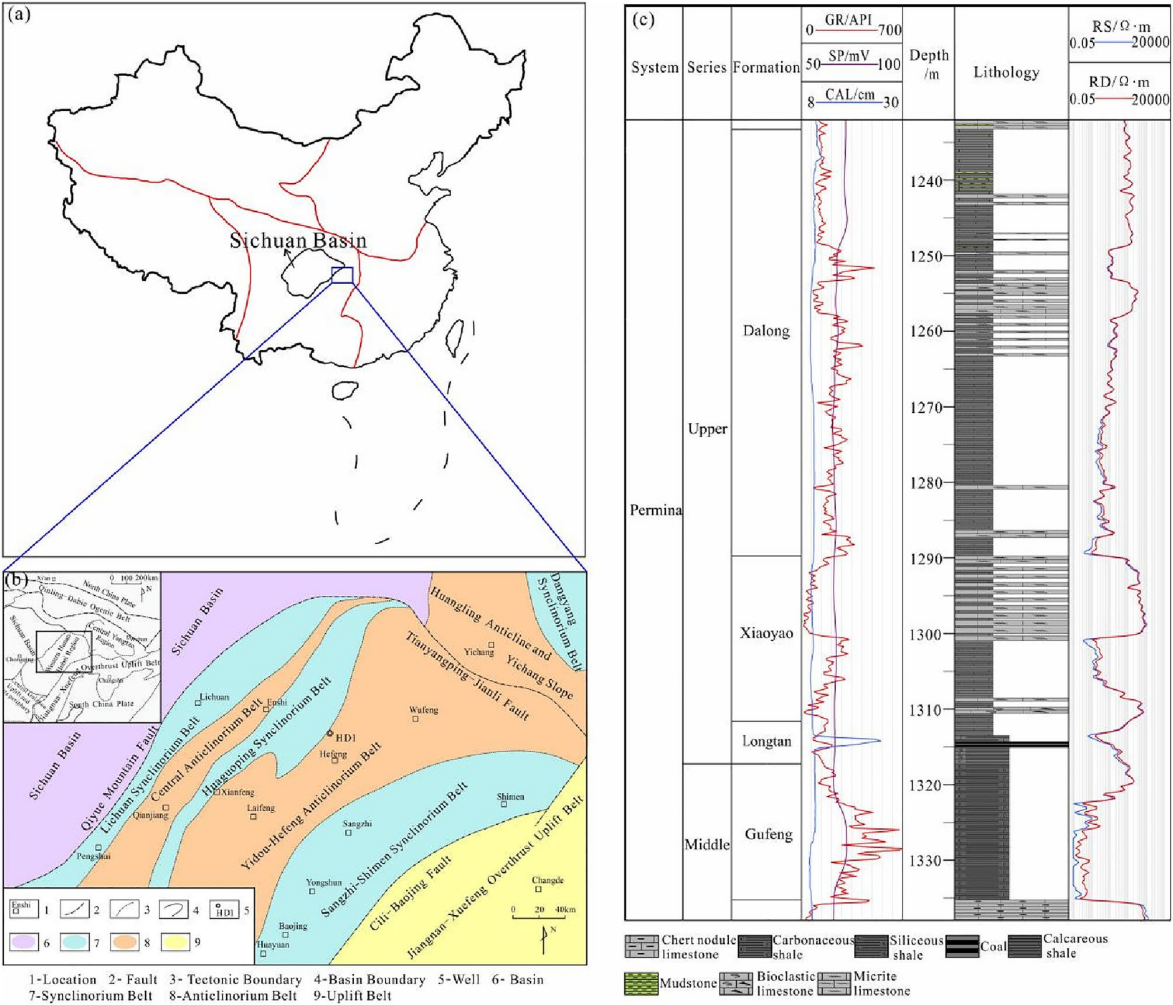
Geographic location, tectonic setting, and lithological column of the Gufeng–Dalong Formation. (**a**) Geographic location of the western Hubei area; (**b**) tectonic setting; (**c**) general lithological column of the Gufeng–Dalong Formation.

Considering the tectonic position, the western Hunan–Hubei fold belt is located in the southeastern part of the Middle and Upper Yangtze plates, which are farther west. The Hunan–Hubei fold belt is an arc-shaped tectonic belt that projects toward the northwest and is jointly controlled by the Qiyueshan and Cili–Baojing deep faults in the NE–NEE direction (Fig. 1); the fold belt is adjacent to the Sichuan Basin in the west, and the northern boundary is the Qinling–Dabie orogenic belt, with the eastern boundary being the Jiangnan–Xuefeng overthrust uplift belt^40^. From southeast to northwest, the western Hunan–Hubei fold belt can be divided into five secondary structural units, namely, the Sangzhi–Shimen synclinorium belt, Yidou–Hefeng anticlinorium belt, Huaguoping synclinorium belt, Central anticlinorium belt and Lichuan synclinorium belt^28,29,37,41^.

Well HD1, which is located in the core of the Chenjiawan syncline, Yidou–Hefeng anticlinorium belt, with a drilled depth of 1366.77 m, reveals that dark shale is present in the Gufeng–Dalong Formation and has a thickness of 102 m. The Gufeng Formation is composed of black carbonaceous shale, gray–black carbonaceous siliceous shale and interbedded carbonaceous shale; the thicknesses are 18–21 m, the fractures are relatively developed and they are mostly filled with calcite. The lithology of the Longtan Formation is black carbonaceous shale with a thin coal seam and gray pyrite-bearing clay rock with carbonaceous shale, with thicknesses of 5.5–6.7 m. The carbonaceous shale and calcareous shale of the Xiayao Formation are developed in the middle of this formation, with thicknesses of 20.7–21.5 m, and the upper and lower parts are mainly limestone. The lithology of the Dalong Formation is mainly gray–black siliceous shale and carbonaceous shale, with thicknesses of 47.5–59.5 m.

## Results and analysis

### Geological characteristics of the reservoir

#### Organic geochemistry

**Figure 2:**
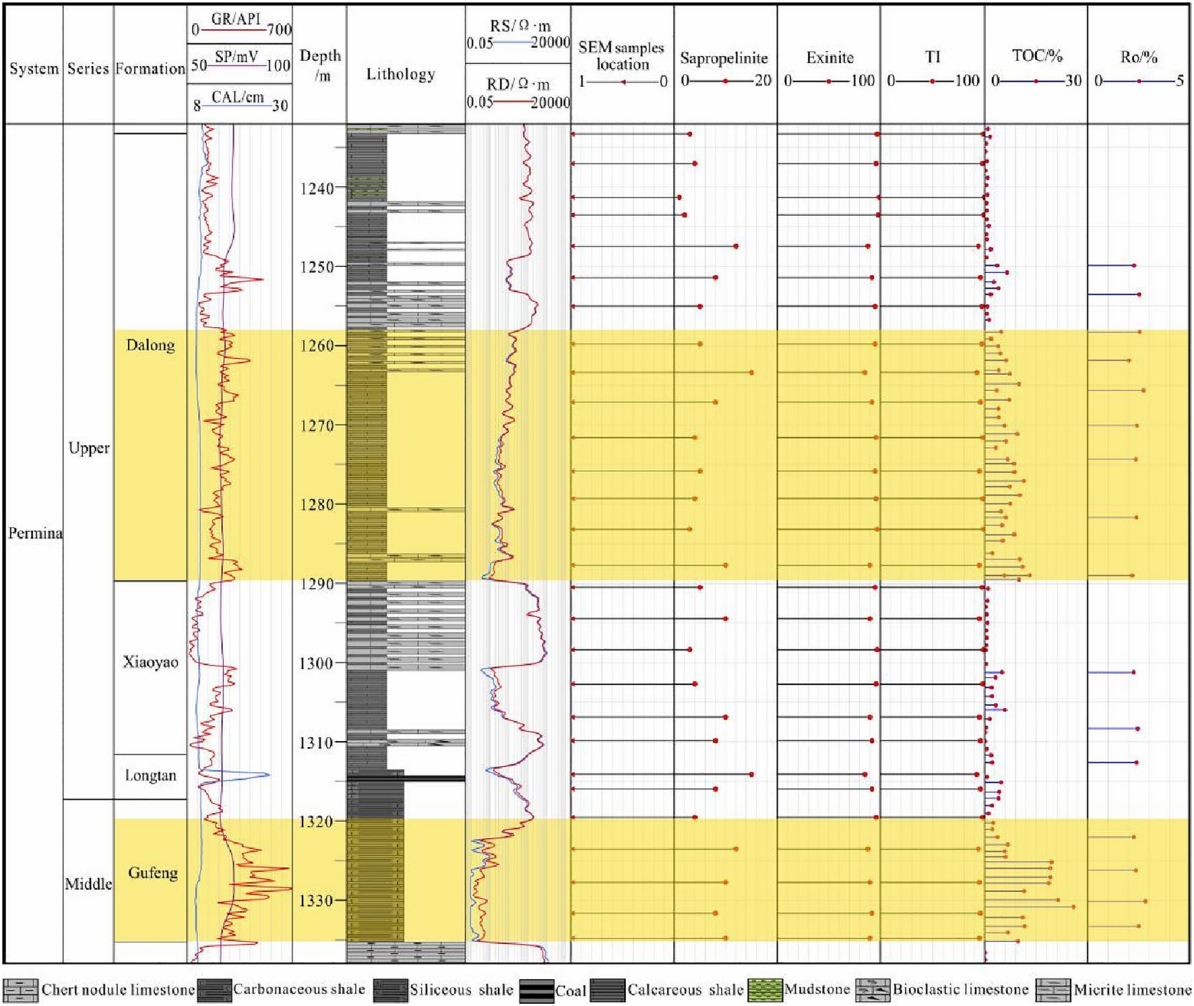
Comprehensive histogram of lithology and organic geochemical indices of the Gufeng–Dalong Formation in well HD1.

The results of kerogen maceral identification show that the maceral composition of the Gufeng–Dalong Formation shale in the HD1 well is predominantly sapropelite, accounting for 85–99%, followed by exinite contents, and it does not contain vitrinite and inertinite. According to the calculated TI values ranging from 92.5 to 99.5 (Fig. 2), the kerogen of the Gufeng–Dalong Formation shale is chiefly type I.

The Ro ranges from 2.01 to 2.81%, with an average of 2.38%, suggesting that the Gufeng–Dalong Formation shale is in a highly overmature stage with predominantly dry gas generation. The distribution of Ro is stable along the profile, with only small changes (Fig. 2).

The TOC content of the Gufeng–Dalong Formation shale varies widely, ranging from 0.1 to 28.93%, with an average value of 5.08%, and the samples with TOC contents greater than 2.0% account for 62% of the total samples. The TOC contents of the Gufeng Formation range from 1.16 to 25.93%, with an average of 10.78%. The Longtan Formation has values of 0.74–4.82%, with an average of 3.21%. The values of the Xiayao Formation are 0.1–18.8%, with an average of 2.35%. The Dalong Formation values are 0.2–13.07%, with an average of 4.49%.

#### Reservoir properties

**Figure 3:**
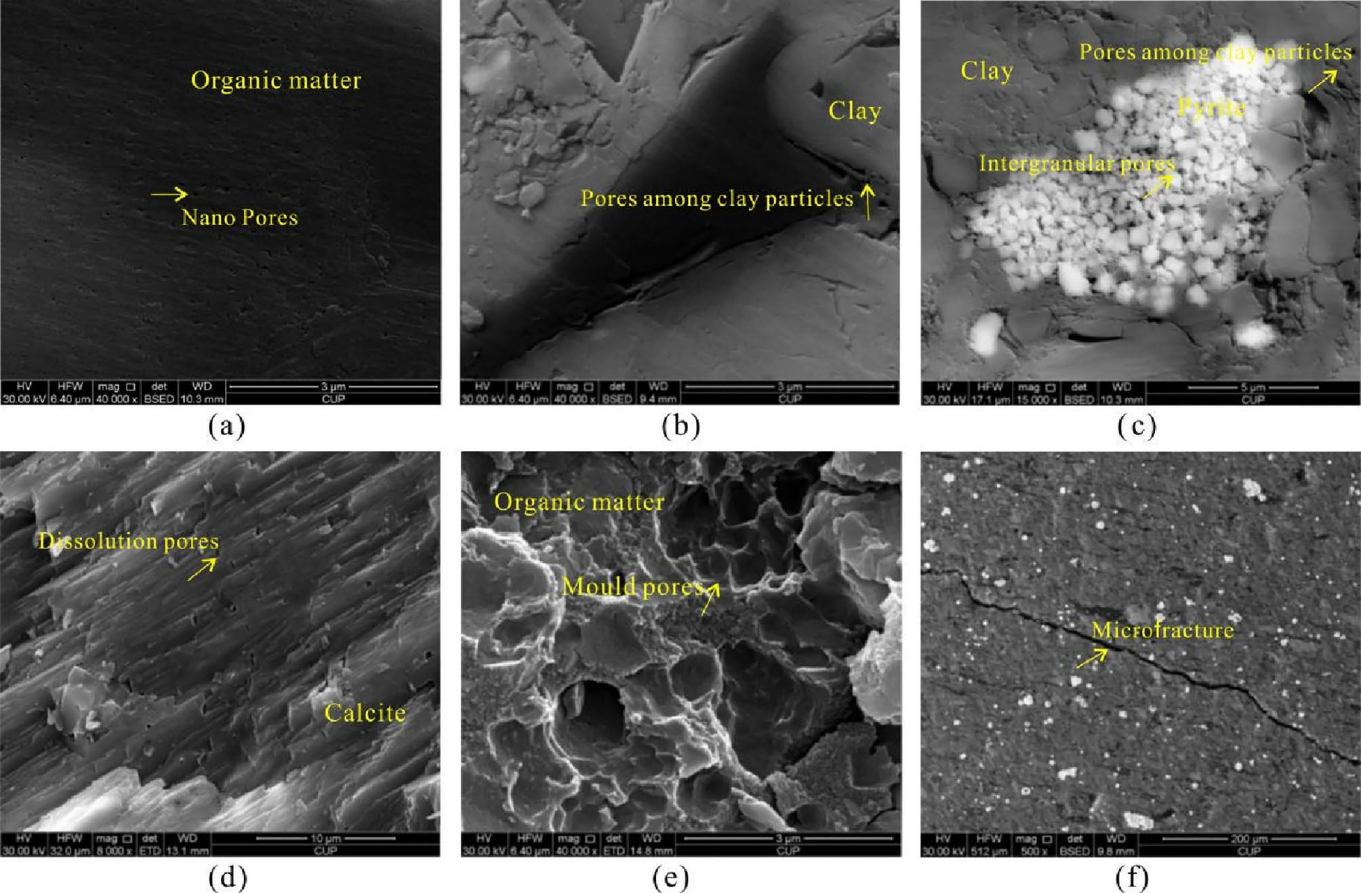
The distribution of pores and fractures of the Gufeng–Dalong Formation shale in the HY1 well. (**a**: 1251.5 m, Dalong Formation, nano-pores in organic matter, ×40,000; **b**: 1343.6 m, Dalong Formation, pores among clay particles, ×10,000; **c**:1319.6 m, Gufeng Formation, pyrite and its intergranular pores, pores among clay particles, ×15,000; **d**: 1327.7 m, Gufeng Formation, dissolution pores in calcite, × 8000; **e**: 1331.6 m, Gufeng Formation, mold pores in an organic matter surface, ×40,000; b:1343.6 m, Dalong Formation, pores among clay particles, ×10,000; **f**: 1279.4 m, Dalong Formation, microfracture, ×500).

Three types of pore structures are developed in the Dalong–Gufeng Formation: organic matter pores, inorganic pores and microfractures (Fig. 3). The organic matter pores are well developed (Fig. 3a), some of which exist between pyrite crystals as intergranular pores. Due to the depletion of organic matter filler in the later thermal evolution process, pyrite-organic matter pores are formed in circular, elliptical, hexagonal and irregular shapes, and complex internal spatial structures in different shapes, such as tubular-column and cave types, are formed. The sizes of organic matter pores vary from nanometers to micrometers. The organic matter pores are mainly developed at the bottom of the Gufeng–Dalong Formation, with a high organic carbon content and well-developed organic matter pores. In general, the development of organic matter pores and their degree of development directly confirms the historical process of hydrocarbon generation in organic matter, which also provides reservoir space for shale gas accumulation. The inorganic pores are mainly pores among clay particles (Fig. 3b), intergranular pores (Fig. 3c), dissolution pores (Fig. 3d), and mold pores (Fig. 3e), the sizes of which are larger overall, with ranges of several hundreds of nanometers to micrometers. A certain number of nano- to micro-fractures developed in the Gufeng–Dalong Formation shales (Fig. 3f). Microfractures exist at the edges of organic matter, with a width of approximately 0.05 μm, and microfractures also exist in the interiors of brittle minerals and between layers of illite and other clay minerals.

The mesopores and micropores were tested by the Barrett‒Joyner‒Halenda (BJH) method, and the results showed that the surface areas (SAs) range from 4.281 to 23.1 m^2^/g, with an average value of 8.58 m^2^/g; the BJH average pore size (APS) values range from 3.82 to 4.058 nm, with an average value of 3.878 nm; and the pore volumes (PVs) are in the range of 4.925–22.23 × 10^–3^ cm^3^/g, with an average value of 11.78 × 10^–3^ cm^3^/g from 5 samples in the Dalong Formation. The SA, APS and PV of the sample from a depth of 1309.65 m in the Xiayao Formation are 11.746 m^2^/g, 3.842 nm and 16.88 × 10^3^ cm^3^/g, respectively. The SA and PV are larger and the APS is slightly smaller than that of the sample located at a depth of 1299.31 m; the values of SA, PV and APS are 1.866 m^2^/g, 4.012 nm and 5.482 × 10^3^ cm^3^/g, respectively; two samples from depths of 1331.26 m and 1321.13 m in the Gufeng Formation are tested; the SA, APS and PV values at a depth of 1331.26 m are 28.129 m^2^/g, 3.842 nm and 22.94 × 10^3^ cm^3^/g, respectively; the SA and PV are larger than those at depths of 1321.13 m, with values of 8.817 m^2^/g and 22.94 × 10^3^ cm^3^/g, respectively; the APS is approximately similar to that of 1321.13 m, with a value of 3.842 nm (Table 1).

**Table 1:** Pore structure parameters of Dalong–Gufeng Formation shales.

Formation	Depth/m	SA/m^2^/g	APS/nm	PV/10^3^ cm^3^/g
Dalong	1246.26	5.308	4.058	7.964
	1258.69	4.281	3.83	11.23
	1268.13	4.673	3.833	8.719
	1277.43	23.1	3.847	22.23
	1288.40	2.276	3.82	4.925
Xiayao	1299.31	1.866	4.012	5.482
	1309.65	11.746	3.842	16.88
Gufeng	1321.13	8.817	3.825	19.1
	1331.26	28.129	3.842	22.94

In general, the siliceous shale of the Dalong–Gufeng Formation has much larger SA and PV, which provides storage space for adsorbed gas and free gas. According to statistics, the contribution to adsorbed gas from the SA provided by the pore structures of mesopores and micropores is greater than that of pore volumes to free gas^24^. Therefore, the properties of the Gufeng–Dalong Formation shale in well HD are favorable for shale gas adsorption.

The porosity values of the Dalong Formation range from 0.45 to 2.54%, with an average value of 1.69%, and the permeability values range from 0.0003 to 0.2099 mD, with an average value of 0.0088 mD (Table 2). The porosity changes regularly with depth; generally, the reservoir physical properties of the lower part of the Dalong Formation are better than those of the middle and upper parts. The porosity values of the Gufeng Formation range from 1.29 to 2.99%, with an average value of 2.02%, and the permeability values range from 0.0019 to 0.089 mD, with an average value of 0.016 mD. Similar to the Dalong Formation, the lower part of the Gufeng Formation has better reservoir physical properties than the middle and upper parts. According to the statistics, the porosity of 67% of the samples is less than 2.0%, and the permeability of 70% of samples is less than 0.005 mD, which corresponds to the category of low porosity and ultralow permeability. On the basis of having a certain scale of reservoir space and pore-fracture structures, shale reservoirs should have a relatively high permeability or shale fracture system with modifiable conditions^42–44^. Otherwise, shale reservoirs with ultralow porosity and ultralow permeability must be fractured to obtain gas productivity^45^.

**Table 2:** Reservoir properties of Dalong–Gufeng Formation shales (The raw data see Supplementary Information).

Formation	Depth/m	Porosity/%	Permeability/mD
Dalong	1233.5–1252.0	$\frac{1.04-2.54}{1.56(13)}$	$\frac{0.0003-0.0083}{0.0041(13)}$
	1252.0–1258.0	$\frac{1.07-2.34}{1.60(6)}$	$\frac{0.0011-0.006}{0.0041(6)}$
	1258.0–1277.0	$\frac{0.45-2.30}{1.78(15)}$	$\frac{0.0009-0.2099}{0.0175(15)}$
	1277.0–1280.5	$\frac{1.85-2.07}{1.98(4)}$	$\frac{0.004-0.0055}{0.0047(4)}$
	1280.5–1289.5	$\frac{1.25-2.10}{1.65(9)}$	$\frac{0.001-0.0357}{0.006(9)}$
Xiayao	1289.5–1311.6	$\frac{0.97-3.58}{1.90(21)}$	$\frac{0.0004-0.0241}{0.0043(21)}$
Gufeng	1317.3–1326.4	$\frac{1.29-2.40}{1.65(6)}$	$\frac{0.0019-0.0304}{0.0078(6)}$
	1326.4–1335.3	$\frac{1.88-2.99}{2.47(5)}$	$\frac{0.0047-0.089}{0.0233(5)}$

$$\frac{1.04-2.54}{1.46(13)}$$ is $$\frac{\mathrm{Min}-\mathrm{Max}}{\mathrm{Avg}(\mathrm{Num})}$$.

#### Gas-bearing characteristics

**Figure 4:**
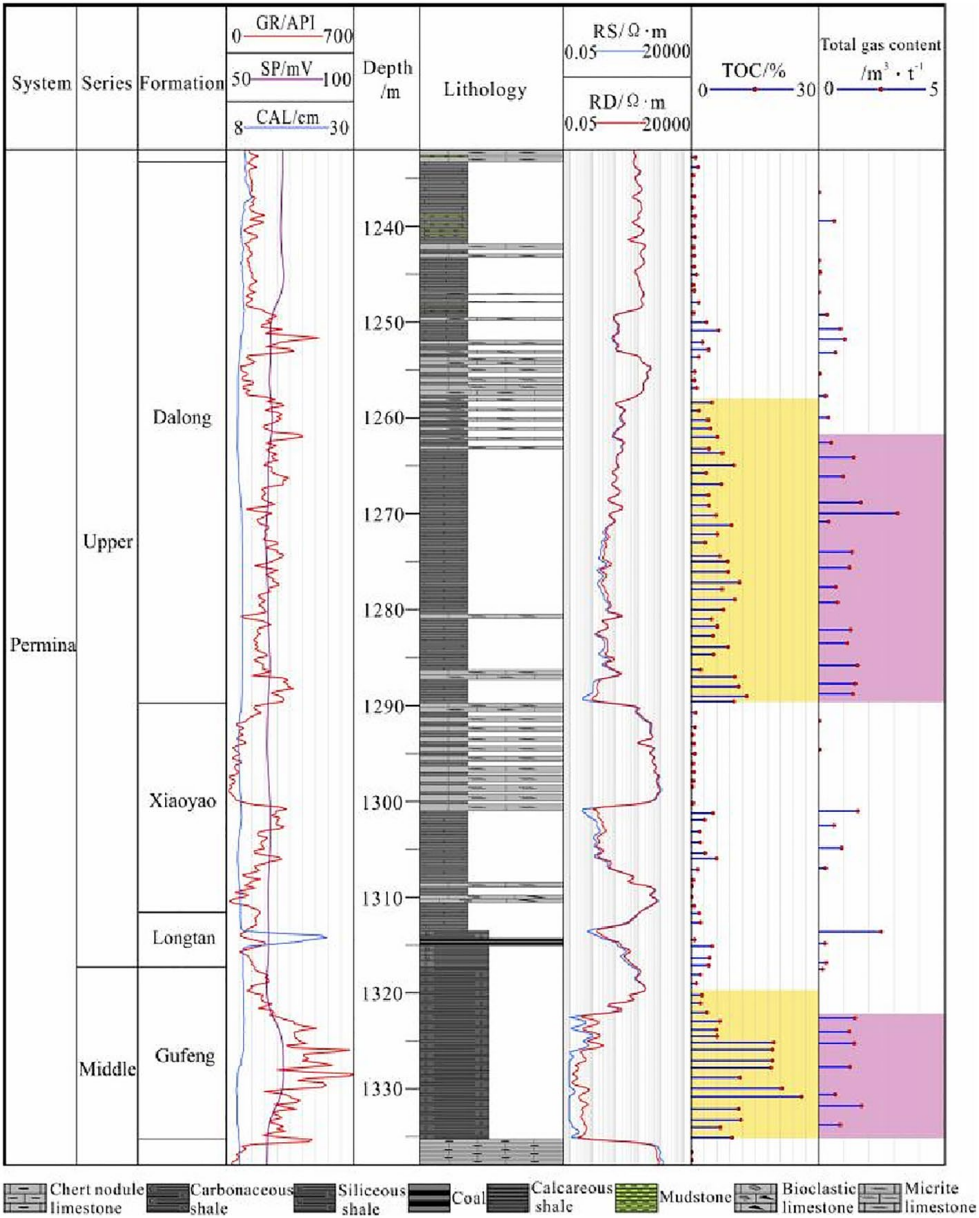
Gas-bearing characteristics of the Gufeng–Dalong Formation in well HD1.

Forty-four samples were taken to perform the field desorption experiment, including 27 samples from the Dalong Formation, 6 samples from the Xiayao Formation and 11 samples from the Gufeng Formation. The results show that the total gas contents range from 0.04 to 3.14 m^3^/t, with an average value of 1.07 m^3^/t. The total gas contents in the Dalong Formation are 0.02–4.39 m^3^/t, with an average of 0.39 m^3^/t. According to the statistics, the total gas contents of 52.6% of the samples are within 1.0–2.0 m^3^/t. The total gas contents of the Xiayao Formation are 0.04–1.97 m^3^/t, with an average of 0.69 m^3^/t. The total gas contents of the Gufeng Formation are 0.21–2.84 m^3^/t, with an average of 1.32 m^3^/t, and the samples with total gas contents ranging from 1.0 to 2.0 m^3^/t account for 55.6% (Fig. 4).

### Characteristics of fracture development

**Figure 5:**
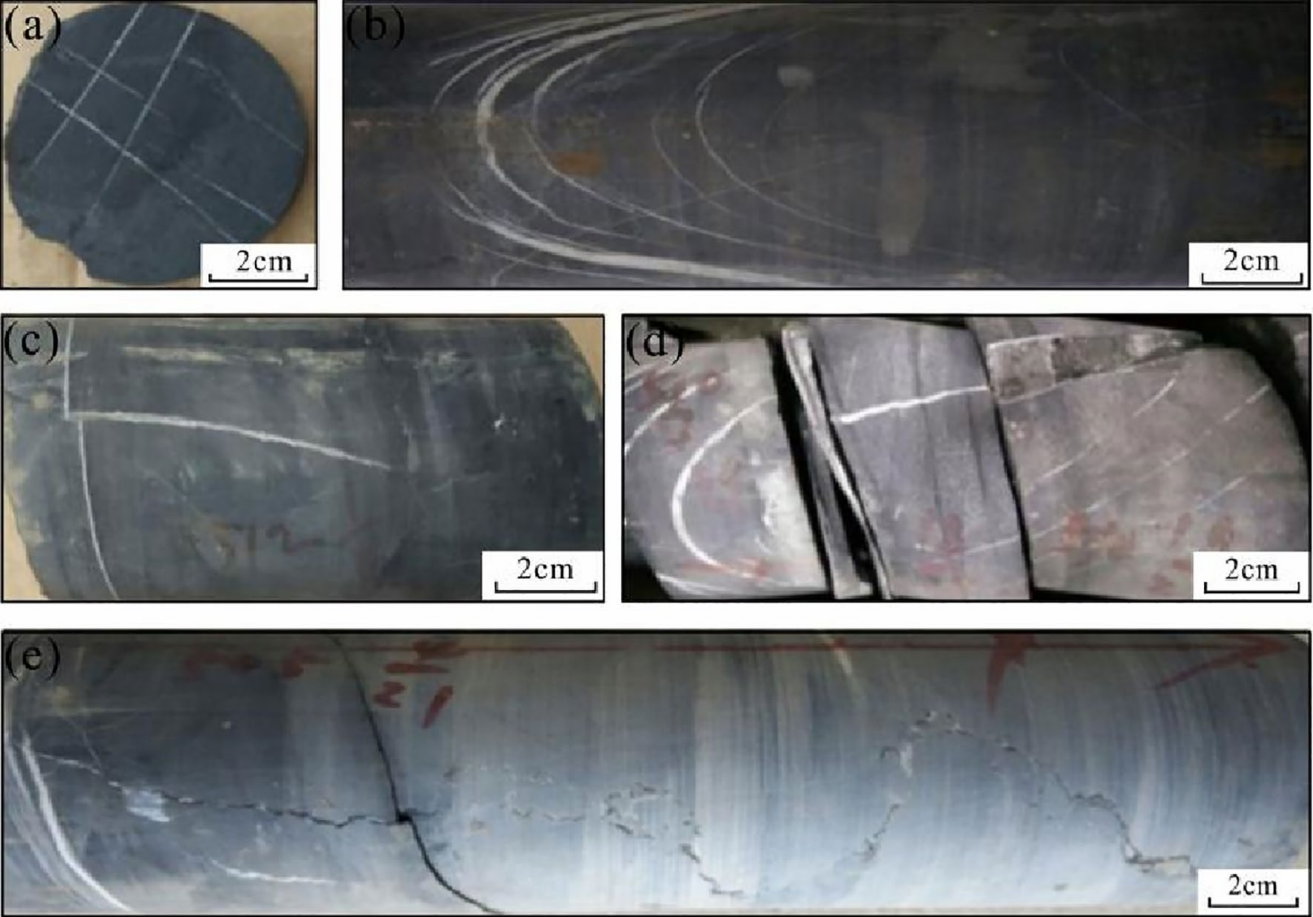
Characteristics of fracture development in well HD1. (**a**: 1267.82 m, gray‒black calcium carbonaceous shale, two groups of orthogonal vertical fractures; **b**: 1258.9–1259.07 m, gray‒black calcium carbonaceous shale with a dark gray thin layer of micrite limestone, parallel high-angle fractures; **c**: 1273.47 m, dark gray calcium carbonaceous shale, vertical fracture cutting a horizontal fracture; **d**: 1269.1 m, gray‒black calcium carbonaceous shale, bedding fractures, the core is easy to crack along the bedding planes; **e**: 1256.15 m, gray limestone with dark gray argillaceous limestone, stylolite fractures).

Shale gas production is directly related to the degree of natural fracture and microfracture development in shale. The existence of fractures and microfractures improves the effectiveness of the hydraulic fracturing effect, thus greatly improving the percolation capacity of shale and providing migration channels for shale gas from bedrock pores to boreholes^42,44,46,47^. The existence of fractures can increase the occurrence of free natural gas and is also helpful for the desorption of adsorbed natural gas. It is also conducive to the connection of nanoscale pores and the formation of a network pore fracture system^48^. For this reason, structural and nonstructural fractures both developed in well HD1. Structural fractures are mainly high-angle/vertical fractures that are mostly filled with calcite, and they are relatively developed in calcareous shale (Fig. 5a and b). Low-angle/horizontal fractures are also developed, the number of which is slightly less than that of high-angle/vertical fractures, and the proportion of filled and unfilled fractures is equivalent (Fig. 5c). Nonstructural fractures are mainly bedding fractures (Fig. 5d) and occasionally visible stylolite structures (Fig. 5e).

The density of natural fractures in the Dalong Formation is generally low, with an average of 4.3 pieces/m, of which the calcareous shale in the upper part of the Dalong Formation is 5.2 pieces/m, and the siliceous shale in the lower part is 4.2 pieces/m. Due to the decrease in the calcium content, the structural fractures are not well developed. The lithology of the Xiayao Formation is mainly carbonaceous limestone, and the density of natural fractures in carbonaceous shale is 9.0 pieces/m, which is relatively high, especially in the upper layer with a relatively high calcium content. The Longtan Formation is mainly composed of carbonaceous limestone, clayey shale and coal seams. The density of natural fractures is generally high, with an average of 7.3 pieces/m. The natural fractures in the black carbonaceous shale in the upper part of the Gufeng Formation are not developed, with a density value of only 2.6 pieces/m, while the fractures of siliceous shale in the lower part are very developed, with a density up to 13.3 pieces/m. The relatively high proportion of natural fractures in each formation is vertical/high-angle filled fractures, followed by horizontal/low-angle filled fractures, horizontal/low-angle unfilled fractures and vertical/high-angle unfilled fractures (Table 3).

**Table 3:** Statistics of natural fractures in the Gufeng–Dalong Formation, well HD1.

Formation	Lithology	Depth/m	vertical/high-angle fractures		horizontal/low-angle fractures		Total/ pieces	Density/ pieces/m
			Filled/pieces	Unfilled/pieces	Filled/pieces	Unfilled/pieces		
Dalong	Calcareous shale	1252.77–1258.77	16	2	11	2	31	5.2
	Siliceous shale	1258.77–1291.47	74	9	35	18	136	4.2
Xiayao	Mixed shale	1291.47–1312.77	84	22	43	42	191	9.0
Longtan	clayey shale, coal seams	1312.77–1318.77	21	4	6	13	44	7.3
Gufeng	Mixed shale	1318.77–1327.77	2	3	7	11	23	2.6
	Siliceous shale	1327.77–1336.77	40	25	20	35	120	13.3
Total	Sum	1252.77–1336.77	237	65	122	121	545	Average: 6.5
	Proportion		43.50%	11.90%	22.40%	22.20%		

In general, the natural fractures in the lower part of the Dalong Formation shale are not well developed, and the natural fracture densities of the Xiayao and Longtan Formations are generally high. The natural fractures in the upper part of the Gufeng Formation carbonaceous shale are not developed, while the natural fractures in the lower part are well developed. The development of natural fractures in the Dalong–Gufeng Formation is favorable for shale fracturing, and the favorable layers of fracturing are mainly distributed in the local layers of the Dalong Formation, Longtan Formation, Xiayao Formation and the lower part of the Gufeng Formation.

### Petrological characteristics

**Figure 6:**
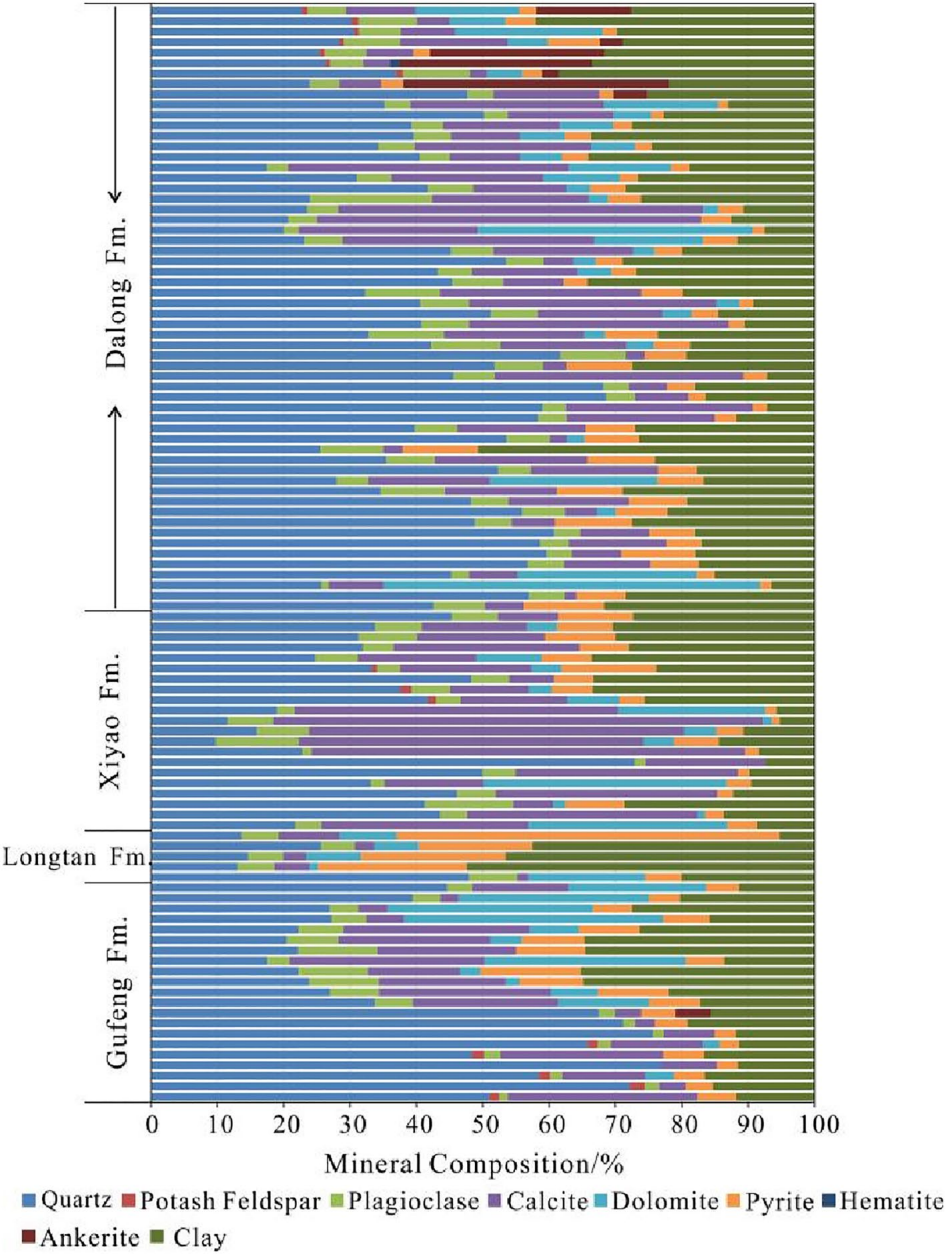
Mineral composition of the Gufeng–Dalong Formation shale in well HD1.

Brittle minerals have an important influence on whether a large number of fracture systems could be produced after shale fracturing, thus forming an effective reservoir for shale gas accumulation^49,50^. The higher the brittle mineral content is, the stronger the supporting effect of the rock skeleton on the pores, and the easier it is to form fractures under external forces^44^. Combined with shale gas exploration, the content of brittle minerals should be greater than 40%^51^. According to the XRD data, the Gufeng–Dalong Formation shale of well HD1 is dominated by quartz and feldspar, with values ranging from 20.7 to 73.0%, with an average value of 47.81%; followed by carbonate minerals (calcite + dolomite) ranging from 1.7 to 75.2%, with an average value of 27.0%; and clay minerals in the range of 2.8–52.4%, with an average value of 21.0%. In addition, pyrite is generally developed, with contents ranging from 0 to 57.7%, with an average of 6.5%. A few samples also contain hematite and ankerite, with small contents (Fig. 6).

For shale with a complex mineral composition, the content of brittle minerals can also be expressed by the mineral brittleness index. Different scholars have different definitions of effective brittle minerals. Zhang et al.^52^ defined quartz, feldspar, carbonate minerals and pyrite as brittle minerals. He et al.^53^ took quartz and clay minerals as representative mineral components of brittle and nonbrittle minerals, statistically studied the subordination between other mineral components and quartz and clay minerals, and then determined effective brittle minerals. Lai et al.^54^ comprehensively considered quartz, feldspar and pyrite as effective brittle minerals when studying the brittleness index of the Niutitang Formation shale in the Cengong area, Guizhou. Quartz is an important mineral for shale brittleness evaluation, with the characteristics of a high elastic modulus, low Poisson's ratio and low toughness. The brittle mineral index is calculated based on the formula defined by Jarvie^55^ in this paper.

$$BRIT = \frac{{V_{{Q{\text{uartz}}}} }}{{V_{{Q{\text{uartz}}}} + V_{Carbonate} + V_{Clay} }}$$

where *BRIT* is the brittle mineral index, and it is dimensionless; *V*_*Quartz*_, *V*_*Carbonate*_, and *V*_*Clay*_ are the percentages of quartz, carbonate and clay minerals, in %, respectively.

**Figure 7:**
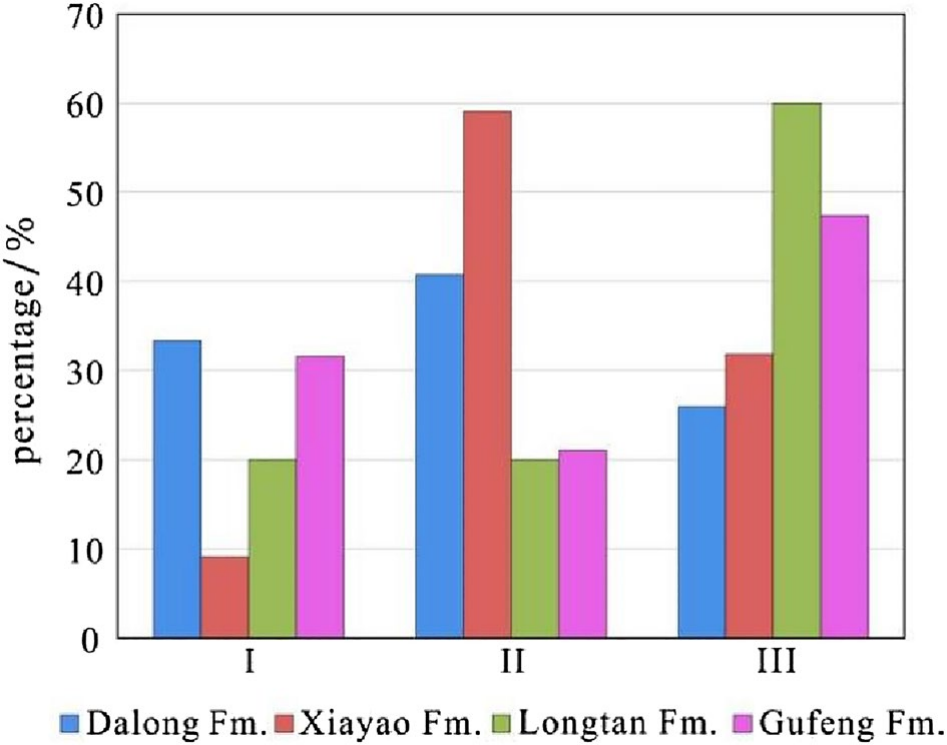
The statistics of the brittle mineral index of the Gufeng–Dalong shale in well HD1.

According to the calculation, the brittle mineral index values of the Gufeng–Dalong Formation shales in well HD1 range from 12.1 to 79.6%, with an average of 44.5%. According to the statistics, the brittle mineral index of 40% of the samples is greater than 50%. The brittle mineral index values of the Dalong Formation range from 18.5 to 74.2%, with an average of 47.3%. Taking a brittle mineral index greater than 55% for class I, ranging from 35 to 55% for class II, and less than 35% for class III as the evaluation standard, the brittle mineral index values of 33.4% of the samples in the Dalong Formation are greater than 55%; 40.7% of the samples have values between 35 and 55%; and 25.9% of the samples have values less than 35% (Fig. 7). The layer with a relatively high brittle mineral index is located in the bottom part of the Dalong Formation at depths from 1249.4 to 1289.5 m, corresponding to the layer with a high organic carbon content and good reservoir physical properties. The brittle mineral index values of the Xiayao Formation range from 12.1 to 74.1%, with an average of 37.9%. A total of 9.1% of samples in the Xiayao Formation are greater than 55%, nearly 60% of samples are between 35 and 55%, and 31.8% of samples are less than 35%. The brittle mineral index values of the Longtan Formation range from 18.1 to 55.0%, with an average of 32.6%. The Longtan Formation is mainly composed of coal seams and carbonaceous shale, the brittle mineral index is low in general, and nearly 60% of the samples are less than 35%. The brittle mineral index values of the Gufeng Formation range from 19.3 to 79.6%, with an average of 46.3%. A total of 52.7% of the samples in the Gufeng Formation have values greater than 35%. Vertically, the layer with a relatively high brittle mineral index is located at depths of 1325.9–1335.3 m in the lower part of the Gufeng Formation. Overall, the brittle mineral index of the Gufeng–Dalong Formation shale is relatively high, and the fracability is good.

### Rock mechanics characteristics

**Figure 8:**
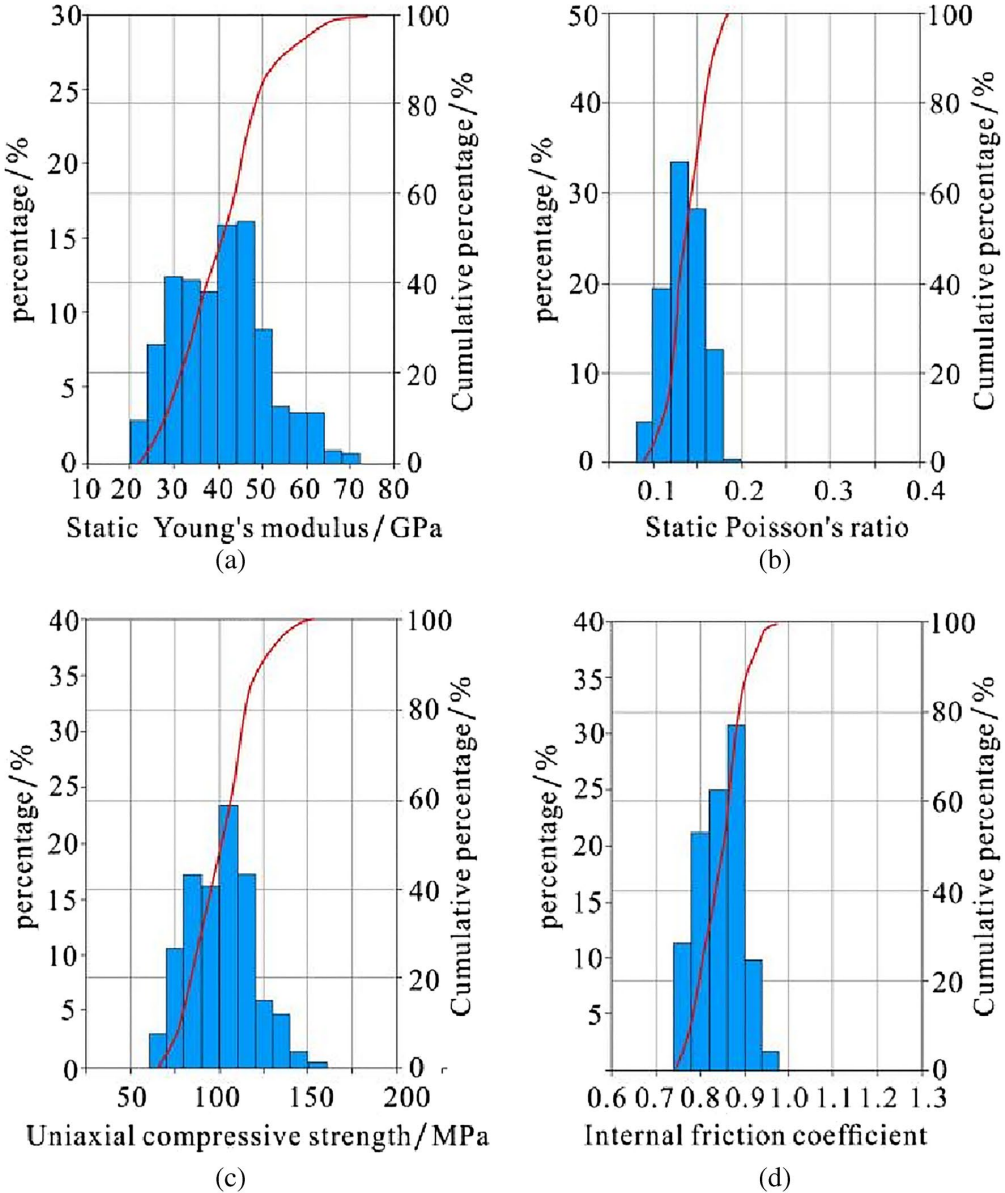
Histogram of the static Young's modulus (**a**), static Poisson's ratio (**b**), uniaxial compressive strength (**c**) and internal friction coefficient (**d**) of the Dalong Formation in well HD1.

Rock mechanics parameters provide basic technical support for fracturing design and are an important basis for fracturing layer selection. The higher the Young's modulus and the smaller the Poisson's ratio of the rock, the better the brittleness. It is generally believed that rock with a Young's modulus greater than 20 GPa and Poisson's ratio less than 0.25 is relatively ideal for brittleness^36,56,57^. The rock mechanics parameters have a good correlation with the acoustic transit time and bulk density of well logging curves. Therefore, the dynamic Poisson's ratio, dynamic Young's modulus, uniaxial compressive strength and internal friction coefficient can be calculated by establishing a statistical model. According to the relational model between the dynamic Young's modulus and static Young's modulus, dynamic Poisson's ratio and static Poisson's ratio, the dynamic data can be converted into static data. According to the processing results of Schlumberger interpretation software, the static Young's modulus of the Dalong Formation is mainly distributed in the range of 26–62 GPa, the static Poisson's ratio mainly ranges from 0.11 to 0.17, the uniaxial compressive strength is mainly distributed in the range of 66–135 MPa, and the internal friction coefficient is mainly distributed in the range of 0.76–0.92. In general, the rock mechanics parameters of the Dalong Formation have the characteristics of good brittleness and high rock strength (Fig. 8).

**Figure 9:**
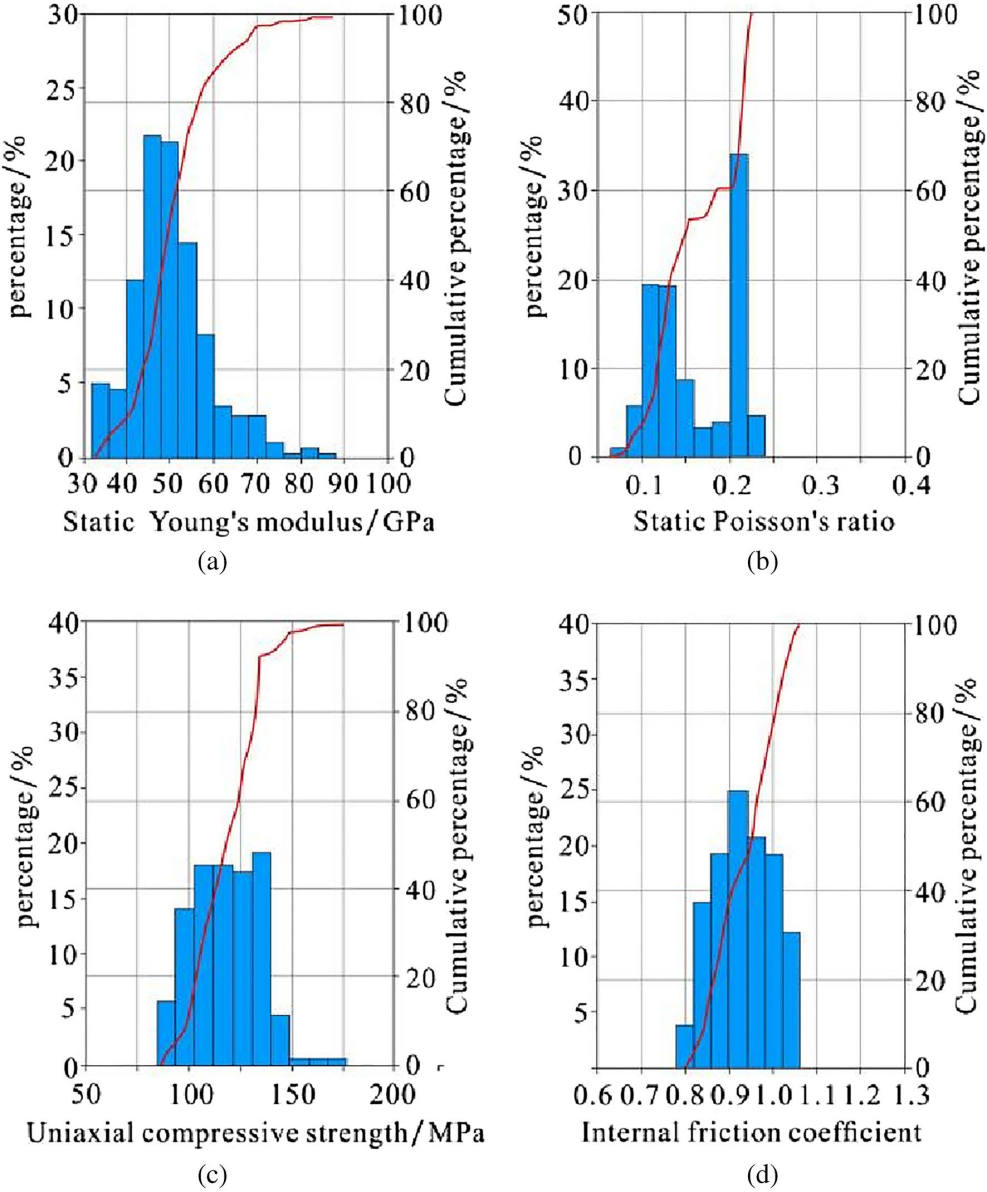
Histogram of the static Young's modulus (**a**), static Poisson's ratio (**b**), uniaxial compressive strength (**c**) and internal friction coefficient (**d**) of the Xiayao Formation in well HD1.

The static Young's modulus values of the Xiayao Formation are mainly distributed in the range of 34–70 GPa, the static Poisson's ratio values mainly range from 0.10 to 0.23, the uniaxial compressive strength values are mainly distributed in the range of 89–143 MPa, and the internal friction coefficient values are mainly distributed in the range of 0.8–1.0. In general, the rock mechanics parameters of the Xiayao Formation have the characteristics of good brittleness and relatively high rock strength (Fig. 9).

**Figure 10:**
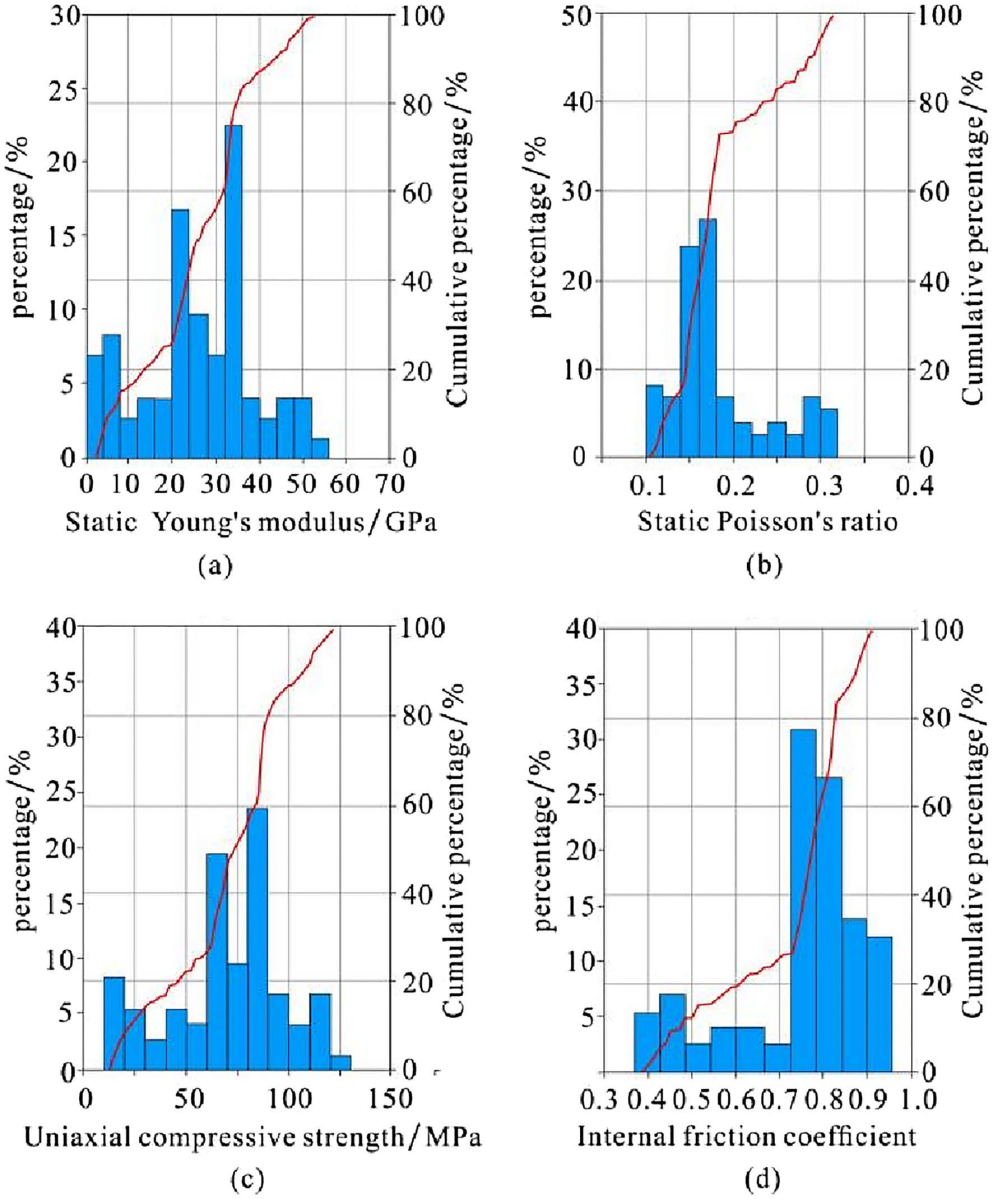
Histogram of the static Young's modulus (**a**), static Poisson's ratio (**b**), uniaxial compressive strength (**c**) and internal friction coefficient (**d**) of the Longtan Formation in well HD1.

The static Young's modulus values of the Longtan Formation are mainly distributed in the range of 2.4–50 GPa, the static Poisson's ratio values mainly range from 0.11 to 0.31, the uniaxial compressive strength values are mainly distributed in the range of 15–113 MPa, and the internal friction coefficient values are mainly distributed in the range of 0.40–0.87. The high carbonaceous shale part of the Longtan Formation has characteristics of poor brittleness and low rock strength, while the other part has characteristics of good brittleness and relatively high rock strength (Fig. 10).

**Figure 11:**
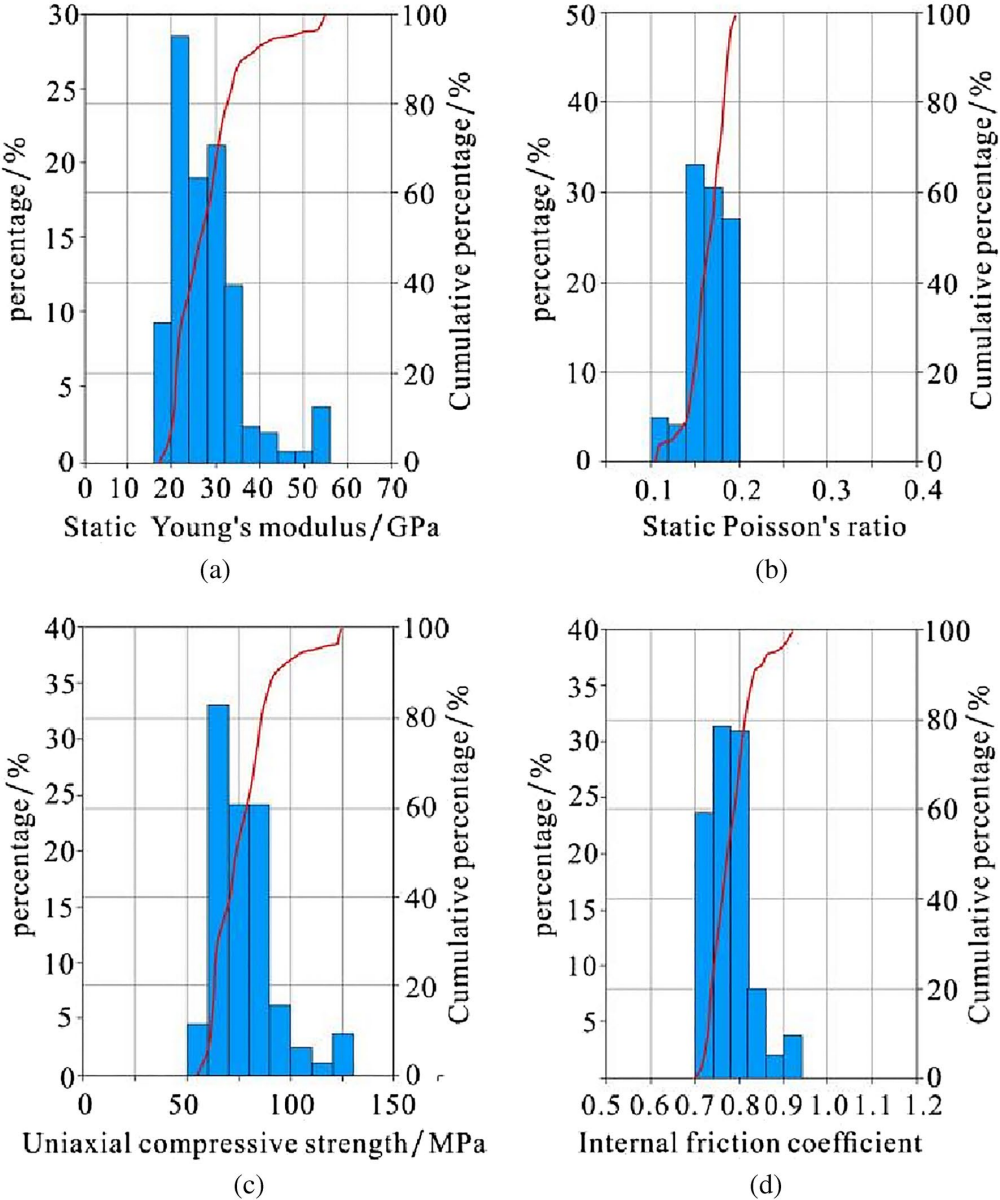
Histogram of Static Young's modulus (**a**), static Poisson's ratio (**b**), uniaxial compressive strength (**c**) and internal friction coefficient (**d**) of the Gufeng Formation in well HD1.

The static Young's modulus values of the Gufeng Formation are mainly distributed in the range of 18–42 GPa, the static Poisson's ratio values mainly range from 0.11 to 0.19, the uniaxial compressive strength values are mainly distributed in the range of 56–125 MPa, and the internal friction coefficient values are mainly distributed in the range of 0.72–0.92. The relatively high carbonaceous shale part of the Gufeng Formation has characteristics of poor brittleness and low rock strength, while the other part has characteristics of good brittleness and relatively high rock strength (Fig. 11).

### Two phases of horizontal pressure difference

The lower the two-phase horizontal pressure difference is, the more beneficial it is to rock formation volume transformation^58–60^. The formula for calculating the two-phase horizontal pressure difference coefficient is:

$$K_{h} = \frac{{\sigma_{y} - \sigma_{x} }}{{\sigma_{x} }}$$

where *K*_*h*_ is the two-phase horizontal pressure difference coefficient; *σ*_*y*_ is the maximum horizontal principal stress, in MPa; and *σ*_*x*_ is the minimum horizontal principal stress, in Mpa.

The two-phase horizontal pressure difference coefficients of the Dalong Formation are relatively high, with values ranging from 0.43 to 0.56 (Table 4). The carbonate rock part located at depths of 1290.6–1300.3 m has the characteristics of a poorer two-phase horizontal pressure difference, with a value of 0.17, while other parts have higher two-phase horizontal pressure differences, with values of 0.17 and 0.36, respectively. The lithology of the Longtan Formation is mainly composed of carbonaceous shale and coal seams. The two-phase horizontal pressure difference coefficients of the high carbonaceous shale section located at depths of 1311.1–1315 m are relatively low, with a value of 0.38. The two-phase horizontal pressure difference coefficients of the Gufeng Formation range from 0.45 to 0.48. Except for the carbonate rock layer of the Xiayao Formation and the high carbonaceous shale layer of the Longtan Formation, the difference coefficients of the other layers are all greater than 0.25.

**Table 4:** The statistics of the horizontal pressure difference of the two phases in well HD1.

Formation	Depth/m	Thickness/m	Minimum horizontal principal stress/Mpa	Maximum horizontal principal stress/Mpa	Two-phase horizontal pressure difference/Mpa	Difference coefficient
Dalong	1232.4–1238.7	6.3	23.1	34.9	11.8	0.51
	1240.0–1248.0	8.0	23.2	35.2	12.0	0.52
	1248.0–1253.4	5.4	23.6	35.3	11.7	0.50
	1253.4–1257.6	4.2	22.7	35.5	12.8	0.56
	1257.6–1266.8	9.2	24.2	35.7	11.5	0.48
	1266.8–1268.7	1.9	25.0	35.8	10.8	0.43
	1268.7–1286.4	17.7	24.6	36.1	11.5	0.47
	1286.9–1290.6	3.7	25.2	36.4	11.2	0.44
Xiayao	1290.6–1300.2	9.6	31.2	36.6	5.4	0.17
	1300.2–1308.2	8.0	24.5	36.9	12.4	0.51
	1308.2–1311.1	2.9	27.2	37.0	9.8	0.36
Longtan	1311.1–1315.0	3.9	26.8	37.1	10.3	0.38
Gufeng	1315.0–1319.0	4.0	25.1	37.2	12.1	0.48
	1319.8–1335.9	16.1	25.8	37.5	11.7	0.45

## Discussion

Taking TOC contents greater than 2.0% as the evaluation standard^42^, the organic-rich shale is distributed in the lower part of the Dalong and Gufeng Formations, with thicknesses of 30 m and 15 m, respectively (Fig. 2). It is part of the ultralow-porosity and ultralow-permeability shale reservoir. Microscopic pores and fractures are developed, with a large surface area and pore volume. The section with a high gas content is located at the bottom of the Gufeng Formation and Dalong Formation. The gas contents at the bottom of the Gufeng Formation range from 0.81 to 2.41 m^3^/t, with an average of 1.54 m^3^/t. The gas contents at the bottom of the Dalong Formation are 0.47–4.39 m^3^/t, with an average of 1.53 m^3^/t (Fig. 4). The comprehensive analysis shows that the organic-rich shale of the Longtan–Dalong Formation in well HD1 has the material basis for gas generation.

The fractures of Gufeng-Dalong Formation are mainly high angle and vertical filled fractures, followed by Low angle and horizontal filled fractures. The natural fractures in lower part Dalong Formation are not developed, with the density of 4.2 pieces/m; the natural fractures of Xiayao Formation, Longtan Formation and the lower part of Gufeng Formation are well developed, with the density of 9.0 pieces/m, 7.3 pieces/m and 13.3 pieces/m. The fractures of each Formation has different degree of development, and which is favorable to the formation of a complex fracture network. Meanwhile, the bedding fractures of organic-rich shales in Gufeng-Dalong Formation are relatively well developed, the existence of shale bedding fractures increased the complexity of fracture, and at the same time, on the one hand, it will increase the filtration and reduce the extension of fractures; on the other hand, the fractures may slip between the beddings and resulting in dislocation, the fractures width of dislocation and slip is very small and has large angle direction change, and the proppant cannot pass through effectively, so the extension of the fractures height and length are limited, and which has a certain negative impact on fracturing fractures generation. Therefore, the leakage caused by shale bedding fractures and the influence on fracture height and length extension should be considered comprehensively in fracturing design.

The mineral composition of Gufeng-Dalong Formation are mainly brittle minerals. The maximum quartz content is up to 77%. The samples with brittle mineral index greater than 35% accounts for 67% of the total samples, and the average brittle mineral index is 44.5%. It is revealed that the brittle mineral index of Gufeng-Dalong Formation shale is high and the fracability is good. The favorable shale layers with high brittle mineral index are located at depth of 1249.4–1289.5 m in the lower part of Dalong Formation and depth of 1325.9–1335.3 m in the lower part of Gufeng Formation respectively.

The static Young's modulus of Gufeng-Dalong Formation is ranging from 26 to 70 GPa, the static Poisson's ratio is 0.10–0.31, the uniaxial compressive strength is 15–143 MPa, and the internal friction coefficient is 1.40–1.0. In general, the rock mechanics of Dalong-Gufeng Formation shales are favorable for fracturing with the characteristics of good-better brittleness and high-higher rock strength. But the two phases horizontal pressure difference is generally large, except for the carbonate rock layer of Xiayao Formation and the high carbonaceous shale layer of Longtan Formation, the difference coefficients of other layers are all greater than 0.25. The double wing fracture is easily formed because of the high two-phase horizontal pressure difference, and the direction of the fracture is basically consistent with the direction of the maximum principal stress. Therefore, it is unfavorable to form complex fractures that effectively expand in space.

Based on the analysis herein, combined with the needs of productivity calculations and comprehensive research, the two fractured layers of the middle–upper Permian Gufeng–Dalong Formation can be divided into depths of 1249–1289.5 m in the lower part of the Dalong Formation and depths of 1300–1335.3 m in the lower part of the Xiayao Formation, Longtan Formation and Gufeng Formation. These two fractured layers include the organic-rich and high gas-content shales in the middle–upper Permian strata.

## Conclusion

The fracability the middle–upper Permian marine shale reservoir in well HD1, Western Hubei Area were systematically evaluated in the paper. The findings of this study demonstrated that the organic-rich shale developed in lower part of Gufeng and Dalong Formation, having the characteristics of low porosity and ultra-low permeability, the micro pores and fractures developed, with larger specific surface area, total pore volume and higher total gas content. The natural fractures undeveloped in lower part of Dalong Formation with the lower linear density, while which well developed in Xiayao, Longtan and the lower part of Gufeng Formation, and the interlayer bedding fractures relatively developed. The Gufeng-Dalong Formation shale also have characteristics of high mineral brittleness index, high static Young’s modulus, low static Poisson’s ratio, and high horizontal pressure difference coefficient of two phases. It is concluded that the shale reservoir of Middle-Upper Permian is favorable for fracturing developed in the lower part of Dalong Formation with the depth of 1249–1289.5 m and the lower part of Xiayao, Longtan and Gufeng Formation with the depth of 1300–1335.3 m.

## Samples and methods

Shale samples of the Gufeng–Dalong Formation at depths from 1233.3 to 1335.3 m were collected from the HD1 well and tested to analyze their organic geochemical features, mineral composition, pore structures and gas-bearing properties.

The experimental data of organic geochemical features include the total organic carbon content (TOC), Rock–Eval pyrolysis, kerogen maceral identification, and sedimentary rock vitrinite reflectance data. A total of 103 samples were tested for TOC analysis following the Chinese National Standard GB/T19145-2003. Twenty-five samples were used for Rock–Eval pyrolysis and kerogen maceral identification following the Chinese National Standards GB/T 18602-2012 and SY/T5125-2014. Following the Chinese National Standard SY/5124-2012, the bitumen reflectance (Br) of 16 samples was measured, and the bitumen reflectance was then converted into the equivalent vitrinite reflectance (Ro) according to the equation Ro = 0.618Br + 0.4.

Mineral compositions were measured by X-ray diffraction (XRD), adhering to the Chinese Oil and Gas Industry Standard SY/T 5163-2010, and the whole-rock mineral and clay mineral compositions of the 103 samples were comprehensively and quantitatively measured. The porosity and permeability of 79 samples were measured based on the GB/T29172-2012 standard.

The pore structures of 28 samples were measured by scanning electron microscopy (SEM) following the SY/T 5162-2014 standard, and the mineral composition, organic matter, pores, and fractures of different sizes were identified with magnifications ranging from 1000 to 80,000. The pore size and specific surface area of 9 samples were quantitatively measured following the SY/T 6154-2019 standard.

The total gas content was measured by field analysis of 44 samples based on the SY/T 6940-2013 standard. The gas content is composed of three parts: the lost gas volume recovered from the core lifting to the sample filling, the analytical gas volume obtained from the analysis after the sample canning, and the residual gas volume remaining in the core determined by grinding the core at a high temperature after terminating the analysis. The corresponding test of the desorption gas components was carried out following the GB/T 13610-2014 standard.

The abovementioned experimental tests were all performed by SGS Unconventional Petroleum Technical Testing (Beijing) Co., Ltd.

### Supplementary Information

Supplementary Information.

## Data Availability

The datasets used and/or analyzed in this study are available from the corresponding author upon reasonable request.
