# Supplementary material for: Fracability evaluation of the middle–upper Permian marine shale reservoir in well HD1, western Hubei area
Source: Sci Rep. 2023 Aug 31;13:14319. doi: 10.1038/s41598-023-40735-z (PMC10471766; doi:10.1038/s41598-023-40735-z)
Supplement: Supplementary file 1 — Supplementary Information. [file 41598_2023_40735_MOESM1_ESM.docx]

Table 1, table 3 and table 4 generated or analysed during this study are included in this published article.

The raw data of table 2 generated or analysed during this study are included in supplementary information files

The raw data of table 2

| Number | Formation | ID | Depth/m | porosity/% | Permeability/mD |
| --- | --- | --- | --- | --- | --- |
| 1 | Dalong Formation | HD1-DL-K1 | 1233.01~1233.11 | 1.48 | 0.0055 |
| 2 |  | HD1-DL-K2 | 1234.38~1234.50 | 2.54 | 0.0034 |
| 3 |  | HD1-DL-K3 | 1235.50~1235.65 | 1.04 | 0.0003 |
| 4 |  | HD1-DL-K4 | 1236.23~1236.33 | 1.14 | 0.0048 |
| 5 |  | HD1-DL-K5 | 1237.40~1237.54 | 1.36 | 0.0040 |
| 6 |  | HD1-DL-K6 | 1238.39~1238.54 | 2.15 | 0.0083 |
| 7 |  | HD1-DL-K7 | 1240.53~1240.65 | 1.67 | 0.0057 |
| 8 |  | HD1-DL-K8 | 1241.15~1241.31 | 1.55 | 0.0047 |
| 9 |  | HD1-DL-K9 | 1242.79~1242.98 | 1.58 | 0.0021 |
| 10 |  | HD1-DL-K10 | 1244.25~1244.45 | 1.43 | 0.0049 |
| 11 |  | HD1-DL-K11 | 1245.50~1245.66 | 1.67 | 0.0056 |
| 12 |  | HD1-DL-K12 | 1248.09~1248.21 | 1.26 | 0.0015 |
| 13 |  | HD1-DL-K13 | 1250.14~1250.32 | 1.38 | 0.0023 |
| 14 |  | HD1-DL-K14 | 1252.77~1252.90 | 1.32 | 0.0011 |
| 15 |  | HD1-DL-K15 | 1253.63~1253.79 | 2.34 | 0.0050 |
| 16 |  | HD1-DL-K16 | 1254.23~1254.33 | 2.00 | 0.0049 |
| 17 |  | HD1-DL-K17 | 1255.66~1255.77 | 1.34 | 0.0016 |
| 18 |  | HD1-DL-K18 | 1256.39~1256.53 | 1.07 | 0.0060 |
| 19 |  | HD1-DL-K19 | 1257.38~1257.53 | 1.58 | 0.0060 |
| 20 |  | HD1-DL-K20 | 1258.61~1258.77 | 2.13 | 0.0041 |
| 21 |  | HD1-DL-K21 | 1259.51~1259.68 | 2.30 | 0.0029 |
| 22 |  | HD1-DL-K22 | 1261.24~1261.41 | 1.21 | 0.0029 |
| 23 |  | HD1-DL-K23 | 1262.64~1262.77 | 1.98 | 0.0034 |
| 24 |  | HD1-DL-K24 | 1263.23~1263.36 | 2.00 | 0.0009 |
| 25 |  | HD1-DL-K25 | 1265.29~1265.42 | 1.61 | 0.0075 |
| 26 |  | HD1-DL-K26 | 1266.90~1267.01 | 1.72 | 0.2099 |
| 27 |  | HD1-DL-K27 | 1269.10~1269.19 | 1.86 | 0.0038 |
| 28 |  | HD1-DL-K28 | 1270.77~1270.91 | 2.14 | 0.0063 |
| 29 |  | HD1-DL-K29 | 1271.83~1271.96 | 0.45 | 0.0018 |
| 30 |  | HD1-DL-K30 | 1272.51~1272.62 | 2.04 | 0.0042 |
| 31 |  | HD1-DL-K31 | 1273.14~1273.26 | 1.84 | 0.0040 |
| 32 |  | HD1-DL-K32 | 1274.53~1274.66 | 2.24 | 0.0065 |
| 33 |  | HD1-DL-K33 | 1275.22~1275.31 | 1.46 | 0.0021 |
| 34 |  | HD1-DL-K34 | 1276.30~1276.45 | 1.86 | 0.0029 |
| 35 |  | HD1-DL-K35 | 1277.36~1277.49 | 2.07 | 0.0040 |
| 36 |  | HD1-DL-K36 | 1278.47~1278.62 | 1.95 | 0.0041 |
| 37 |  | HD1-DL-K37 | 1279.50~1279.62 | 2.05 | 0.0055 |
| 38 |  | HD1-DL-K38 | 1280.27~1280.43 | 1.85 | 0.0052 |
| 39 |  | HD1-DL-K39 | 1281.15~1281.25 | 1.86 | 0.0022 |
| 40 |  | HD1-DL-K40 | 1282.66~1282.77 | 1.70 | 0.0033 |
| 41 |  | HD1-DL-K41 | 1283.80~1283.92 | 1.63 | 0.0018 |
| 42 |  | HD1-DL-K42 | 1284.34~1284.43 | 1.25 | 0.0357 |
| 43 |  | HD1-DL-K43 | 1285.45~1285.55 | 1.27 | 0.0032 |
| 44 |  | HD1-DL-K44 | 1286.20~1286.35 | 1.83 | 0.0010 |
| 45 |  | HD1-DL-K45 | 1287.52~1287.66 | 1.52 | 0.0013 |
| 46 |  | HD1-DL-K46 | 1288.34~1288.46 | 2.10 | 0.0026 |
| 47 |  | HD1-DL-K47 | 1289.32~1289.45 | 1.65 | 0.0028 |
| 48 | Xiayao Formation | HD1-XY-K1 | 1290.84~1291.01 | 1.48 | 0.0022 |
| 49 |  | HD1-XY-K2 | 1250.81~1253.13 | 1.38 | 0.0016 |
| 50 |  | HD1-XY-K3 | 1285.94~1291.31 | 1.51 | 0.0015 |
| 51 |  | HD1-XY-K4 | 1292.02~1292.15 | 1.02 | 0.0004 |
| 52 |  | HD1-XY-K5 | 1292.85~1292.97 | 1.19 | 0.0021 |
| 53 |  | HD1-XY-K6 | 1294.34~1294.42 | 1.94 | 0.0021 |
| 54 |  | HD1-XY-K7 | 1295.08~1295.22 | 1.80 | 0.0017 |
| 55 |  | HD1-XY-K8 | 1296.73~1296.86 | 1.41 | 0.0030 |
| 56 |  | HD1-XY-K9 | 1297.57~1297.66 | 1.94 | 0.0037 |
| 57 |  | HD1-XY-K10 | 1298.17~1298.32 | 2.15 | 0.0054 |
| 58 |  | HD1-XY-K11 | 1299.27~1299.35 | 1.91 | 0.0241 |
| 59 |  | HD1-XY-K12 | 1300.03~1300.14 | 2.09 | 0.0030 |
| 60 |  | HD1-XY-K13 | 1300.98~1301.06 | 2.54 | 0.0093 |
| 61 |  | HD1-XY-K14 | 1302.19~1302.27 | 1.79 | 0.0041 |
| 62 |  | HD1-XY-K15 | 1304.05~1304.20 | 0.97 | 0.0014 |
| 63 |  | HD1-XY-K16 | 1305.25~1305.35 | 2.31 | 0.0044 |
| 64 |  | HD1-XY-K17 | 1206.62~1206.77 | 3.58 | 0.0035 |
| 65 |  | HD1-XY-K18 | 1307.66~1307.79 | 1.77 | 0.0014 |
| 66 |  | HD1-XY-K19 | 1308.11~1308.27 | 1.98 | 0.0020 |
| 67 |  | HD1-XY-K20 | 1310.45~1310.55 | 2.49 | 0.0065 |
| 68 |  | HD1-XY-K21 | 1311.31~1311.39 | 2.78 | 0.0073 |
| 69 | Gufeng  Formation | HD1-GF-K1 | 1312.42~1312.56 | 2.40 | 0.0058 |
| 70 |  | HD1-GF-K2 | 1317.08~1317.21 | 1.57 | 0.0304 |
| 71 |  | HD1-GF-K3 | 1319.67~1319.75 | 1.37 | 0.0019 |
| 72 |  | HD1-GF-K4 | 1320.57~1320.65 | 1.29 | 0.0025 |
| 73 |  | HD1-GF-K5 | 1322.14~1322.23 | 1.38 | 0.0036 |
| 74 |  | HD1-GF-K6 | 1325.12~1325.22 | 1.88 | 0.0024 |
| 75 |  | HD1-GF-K7 | 1326.93~1327.01 | 2.40 | 0.0890 |
| 76 |  | HD1-GF-K8 | 1327.90~1328.01 | 1.88 | 0.0047 |
| 77 |  | HD1-GF-K9 | 1329.38~1329.53 | 2.41 | 0.0069 |
| 78 |  | HD1-GF-K10 | 1331.19~1331.33 | 2.99 | 0.0100 |
| 79 |  | HD1-GF-K11 | 1333.00~1333.11 | 2.65 | 0.0058 |
| Experimental condition | Confining pressure:2.5MPa, Inlet pressure: 0.5MPa | | | | |
| Standard | SY/T 5336-2006 | | | | |
